# Global phylogeography and microdiversity of the marine diazotrophic photoautotrophs *Trichodesmium* and UCYN-A

**DOI:** 10.1128/msphere.00245-25

**Published:** 2025-07-11

**Authors:** Angie Nguyen, Lucas J. Ustick, Alyse A. Larkin, Adam C. Martiny

**Affiliations:** 1 Department of Biological Chemistry, University of California Irvine School of Medicine, Irvine, California, USA; 2 Department of Ecology and Evolutionary Biology, University of California Irvine, Irvine, California, USA; 3 Department of Earth System Science, University of California Irvine, Irvine, California, USA; Clemson University, Clemson, South Carolina, USA

**Keywords:** nitrogen fixation, microbial ecology, environmental microbiology, marine microbiology, cyanobacteria, phylogeography

## Abstract

Photoautotrophic diazotrophs, specifically the genera *Trichodesmium* and UCYN-A, play a pivotal role in marine nitrogen cycling through their capacity for nitrogen fixation. Despite their global distribution, the microdiversity and environmental drivers of these diazotrophs remain underexplored. This study provides a comprehensive analysis of the global diversity and distribution of *Trichodesmium* and UCYN-A using the nitrogenase gene (*nifH*) as a genetic marker. We sequenced 954 samples from the Pacific, Atlantic, and Indian Oceans as part of the Bio-GO-SHIP project. Our results reveal significant phylogenetic and biogeographic differences between and within the two genera. *Trichodesmium* exhibited greater microdiversity compared to UCYN-A, with clades showing region-specific distribution. *Trichodesmium* clades were primarily influenced by temperature and nutrient availability. They were particularly frequent in regions of phosphorus stress. In contrast, UCYN-A was most frequently observed in regions experiencing iron stress. UCYN-A clades demonstrated more homogeneous distributions, with a single sequence variant within the UCYN-A1 clade dominating across varied environments. The biogeographic patterns and environmental correlations of *Trichodesmium* and UCYN-A highlight the role of microdiversity in their ecological adaptation and reflect their different ecological strategies. These findings underscore the importance of characterizing the global patterns of fine-scale genetic diversity to better understand the functional roles and distribution of marine nitrogen-fixing photoautotrophs.

**IMPORTANCE:**

This study provides insights into the global diversity and distribution of nitrogen-fixing photoautotrophs, specifically *Trichodesmium* and UCYN-A. We sequenced 954 oceanic samples of the *nifH* nitrogenase gene and uncovered significant differences in microdiversity and environmental associations between these genera. *Trichodesmium* showed high levels of sequence diversity and region-specific clades influenced by temperature and nutrient availability. In contrast, UCYN-A exhibited a more uniform distribution, thriving in iron-stressed regions. Quantifying these fine-scale genetic variations enhances our knowledge of their ecological roles and adaptations, emphasizing the need to characterize the genetic diversity of marine nitrogen-fixing prokaryotes.

## INTRODUCTION

Microbial biogeography and phylogenetic diversity are intricately linked, with genetic variations as small as 0.5% translating into significant physiological differences among microorganisms (1). Such genetic differences, referred to as “microdiversity,” can have profound implications for microbial adaptation and ecological functions. In many bacterial species, microdiversity leads to the formation of distinct clades, which are genetically cohesive populations adapted to specific environmental conditions (2). The concept of ecologically distinct clades is well illustrated in the cyanobacterium *Prochlorococcus*, which is divided into multiple clades that are adapted to varying light, temperature, and nutrient conditions in marine environments (3–5). This study extends the exploration of microbial microdiversity to photoautotrophic diazotrophs, focusing on the globally significant genera *Trichodesmium* and *Candidatus Atelocyanobacterium thalassa* (UCYN-A), both of which contribute to marine nutrient cycling by fixing atmospheric nitrogen into bioavailable forms (6).

*Trichodesmium*, a free-living cyanobacterium, is known for its ability to form large colonies and bloom in nutrient-poor, warm oceanic regions, particularly in the Atlantic Ocean and places where temperatures exceed 25°C (7–12). Previous phylogenetic analyses have divided *Trichodesmium* into four clades (*Tricho* I, II, III, and IV), with clades I and III being the most well-documented based on the nitrogenase (*nifH*) gene (13, 14). Clade II, in particular, remains poorly understood due to a lack of comprehensive reference sequences (15). While the isolate IMS101 from clade III has been used in laboratory experiments, its applicability to Earth system models has been questioned, as regional studies have shown this clade to be less abundant than others in the environment (10, 16–19). Despite extensive studies on *Trichodesmium* at the genus level, there is a need for a deeper understanding of the biogeographic distribution and microdiversity of its clades to elucidate their ecological roles and adaptations.

Similarly, *Candidatus Atelocyanobacterium thalassa* or UCYN-A is a widespread obligate symbiont marine diazotroph that has formed symbiotic relationships with eukaryotic hosts, facilitating its role in nitrogen fixation across diverse marine environments (6, 20). Phylogenetic analyses of UCYN-A, based on the *nifH* gene, have identified multiple clades (UCYN-A1 to -A6), each exhibiting distinct geographical distributions and environmental associations (21–23). However, it is still unclear what the exact drivers of UCYN-A abundance are as a genus and how widespread each clade is globally. UCYN-A has become an increasingly significant model organism as it was recently shown to be tightly integrated into its host algal cell, suggesting it is an early evolutionary stage nitrogen-fixing organelle termed a “nitroplast” (24). This highlights the necessity for comprehensive sequencing and analysis to better understand the distribution, diversity, and ecological significance of UCYN-A clades in the global ocean.

To address these gaps in knowledge, we conducted a global biogeographic analysis of the phylogeography and microdiversity of *Trichodesmium* and UCYN-A using *nifH* gene sequencing across 954 new seawater samples from the Pacific, Atlantic, and Indian Oceans, as part of the Bio-GO-SHIP project. By characterizing the phylogenetic diversity and mapping the global distributions of these photoautotrophic diazotrophs, we aim to uncover the environmental drivers and functional differences that shape their ecological niches. This study provides a detailed analysis of the biogeographic patterns and microdiversity of *Trichodesmium* and UCYN-A, contributing to our understanding of their roles in marine ecosystems and informing future research on the adaptation and evolution of marine microorganisms.

## MATERIALS AND METHODS

### Seawater collection

Between 2 and 10 L of surface water was collected from each cruise using the Niskin rosette system or the ship’s circulating seawater system. Samples were filtered through a 0.22 µm pore size Sterivex filter (Millipore, Darmstadt, Germany) and preserved with a lysis buffer (23.4 mg/mL NaCl, 257 mg/mL sucrose, 50 mmol/L Tris-HCl, and 20 mmol/L EDTA) and stored at −20°C before extraction.

### DNA extraction

A total of 180 mL lysozyme buffer (50 mg/L) was added to each Sterivex containing seawater before incubation at 37°C for 30 min. Then, 180 mL proteinase K (1 mg/mL) and 100 mL SDS were added to the filter before overnight incubation at 55°C on a plate shaker. The filtered liquid, along with sodium acetate and cold isopropanol, was divided into microcentrifuge tubes and then incubated for >2 h at −20°C. The samples were then centrifuged at 15,000 × *g* at 4°C for 30 min. A DNA pellet was isolated by disposing of the supernatant liquid. The pellet was then suspended in Tris-EDTA (TE) buffer for 30 min in a 37°C water bath. Following that, the DNA was extracted using a genomic DNA Clean and Concentrator kit (Zymo Corp., Irvine, CA, USA).

### Polymerase chain reaction

The concentration of the extracted DNA samples was measured using a Qubit dsDNA HS assay kit (Life Technologies, Carlsbad, CA, USA). The samples were then diluted to 2 µg/mL with TE buffer. The 2 µg/mL samples were then amplified using a nested PCR optimized for *nifH* according to the (25) protocol. Modifications to the original protocol were based on tests using environmental samples and a gradient of possible reaction conditions. PCRs were done on a C1000 Bio-Rad Thermocycler with 20 µL of PCR mixture including 10 µL AccuStart II PCR Toughmix (QuantaBio, Beverly, MA), 4 µL MilliQ, 1 µL each of forward and reverse primers, and 4 µL diluted DNA. For the primary PCR, the reverse primers were *nifH*3 and *nifH*4, and the DNA template was the extracted DNA. The C1000 Touch Thermo Cycler (Bio-Rad) reaction conditions were 94°C for 1 min, 47°C for 1:30 min, and 72°C for 1:30 min repeated for 30 cycles with a final extension at 72°C for 10 min before holding at 4°C. For the secondary PCR, the reverse primers were *nifH*1 and *nifH*2, and the DNA template was the amplified DNA from the primary PCR. The reaction conditions were 94°C for 1 min, 54°C for 1:30 min, and 72°C for 1:30 min repeated for 30 cycles with a final extension at 72°C for 10min before holding at 4°C. The nested PCR products were then cleaned using a Zymo 96-well Clean and Concentrator Kit (Zymo Corp., Irvine, CA, USA).

### nifH amplicon sequencing

Illumina-specific transposase adapters (i5 and i7) were added to the *nifH* amplicons. The amplicons were mixed with 1 µL of each adaptor as well as PCR mix and run under the following conditions: 10 cycles of 94°C for 1 min, 54°C for 1 min, and 72°C for 1:30 min, and a final extension step at 72°C for 10 min. The concentration of each sample was determined using fluorescence via a gel and a standardized curve of samples of known concentrations (determined using a Qubit dsDNA HS assay kit [Life Technologies, Carlsbad, CA, USA]). After pooling the samples, large fragments were removed using Agencourt AMPure XP beads (Beckman Coulter Inc., Brea, CA). Finally, a Bioanalyzer chip (Agilent, Santa Clara, CA) was used to check the size distribution of the pool before sequencing it at the UCI Genomics Center.

### Sequence analysis

The 954 new *nifH* samples produced in this study (BioProject ID PRJNA656268) were combined with 131 *nifH* amplicon samples from reference 26 in FASTQ format (BioProject ID PRJNA385736) and 17 *nifH* amplicon samples from (8) in FASTQ format (BioProject ID PRJNA328516), resulting in a combined data set of 1102 FASTQ files. Metadata for all FASTQ samples included in the study can be found in Data S3 at https://doi.org/10.5281/zenodo.15682628.

Raw sequence data in FASTQ format were processed using the QIIME 2 pipeline (27) (version 2019.7). First, the data were imported into QIIME2 using the qiime tools import command. Next, primers were removed from the sequences using the qiime cutadapt trim-paired command, with specific forward and reverse primers provided. Sequences were then quality filtered and denoised using the qiime dada2 denoise-paired command, removing low-quality reads and trimming sequences to the appropriate lengths based on pre-defined parameters. A feature table and representative sequences were generated.

The trimmed and quality-filtered nucleotide sequences were translated into protein sequences using the transeq tool from the EMBOSS package (28). The sequences were translated across six reading frames to identify the most complete protein sequences for each operational taxonomic unit (OTU). Ensembl BLAST (29) was used to align protein sequences to a reference database of known *nifH* sequences from the Zehr Lab (https://www.jzehrlab.com/nifh, last updated in June 2017) to identify *Trichodesmium* and UCYN-A sequences.

Across all samples, the 100 most abundant amplicon sequence variants (ASVs) were identified for both *Trichodesmium* and UCYN-A. The top 100 ASVs were combined with curated sequences to contextualize our resulting trees. For *Trichodesmium*, we included *nifH* sequences from *Trichodesmium thiebautii* H9-4 (LAMW01000105.1) and *Trichodesmium* sp. IMS101 (AF167538.1) (30, 31). For UCYN-A, we included *nifH* sequences from NC_013771.1 (32), AF299420.1 (33), amplicon sequences from reference 23, and consensus sequences from reference 21. We aligned and trimmed these reads using MUSCLE 5.1 (34) and trimAL 1.4.1 with the -gappyout argument (35). The best tree model was determined using megaX (36). Based on this analysis, a GTRGAMMA model was selected for both trees. Phylogenetic trees were created using raxml-8.2.12 with the raxmlHPC-PTHREADS-SSE3 command (37) with AY768418.1 as an outgroup. Clade annotations were applied based on the resulting trees. Trees were explored using the ggtree package in R (38, 39).

Metagenomic sequence processing of *nifH* sequences in 937 Bio-GO-SHIP metagenome samples was identified (see reference 40 for additional sample details). Initial quality filtering (qtrim = rl, trimq = 10, minlen = 25, ktrim = r, and *k* = 25) and read merging were done using BBTools (41). Merged and unpaired reads were translated with Prodigal using the “meta” flag (42). The aforementioned *nifH* database was used as a query using BLAT (-fastMap -minIdentity = 20). A reciprocal search was done using the BLAT hits as queries against the *nifH* database supplemented with common non-*nifH* sequences (mainly Proteochlorophyllide Reductase) using BLAST and a threshold of 1e–5. All sequences assigned as *nifH* with BLAST were aligned using hmmer (hmmer.org) and classified using pplacer (43) and GraftM (44).

Due to the rarity of diazotrophs in many plankton communities, their coverage was low in metagenomic libraries. Therefore, 33% of the metagenomic samples had zero identified *nifH* reads. To overcome this metagenomics detection issue, we only used metagenomic samples with a minimum of 10 times coverage of *nifH* and compared them to amplicon samples with a minimum of 100 times coverage of *nifH*. A total of 69 samples in our data set met these quality thresholds. We calculated the Spearman correlation using the R cor.test function for those samples that met these requirements.

### Statistical analysis

All statistical analysis was done using R version 4.2.1 (39). Shannon diversity was calculated on the relative abundance of all ASVs using the “diversity” function from the vegan package version 2.6-2 (45). *t*-Tests were made using stat_compare_means from the ggpubr package (46). Our data are continuous and represent a random sampling of a population (tricho vs ucyn-a). Because the data sets are similar in size (*n* = 810 and 574), the independent samples *t*-test is robust to differences in SD. Bray-Curtis distances were calculated on the relative abundances of all ASVs using the “vegdist” function, and the resulting PCoA decomposition was done using the “wcmdscale” function from the vegan package (45). Permutational multivariate analysis of variance (PERMANOVA) analysis was performed using “adonis2” from the vegan package. Correlations between environmental parameters and the PCoA were calculated using “envfit” from the vegan package (45). Spearman correlations were calculated on the relative abundances of *Trichodesmium* and UCYN-A along with each clade using the cor.test function from the base R stats package (39). Nutrient stress values were obtained from (47). These measurements have previously been linked to surface nutrient concentrations and proposed as an indicator of nutrient limitation (47–49). We used Ω P high, Ω Fe medium, and Ω N high, which are derived from the abundance of the following genes in metagenomes: alkaline phosphatase genes (phoA and phoX; Ω P), Fe uptake transporter genes (cirA, expD, febB, fepB/C, tolQ, and tonB; Ω Fe), and nitrite and nitrate assimilation and uptake genes (focA, moaA-E, moeA, napA, narB, and nirA; Ω N). While these values are based on *Prochlorococcus* populations (non-diazotrophs), the indices have been shown to be accurate bio-indicators of the full community state (47, 49).

### Figure generation

Phylogenetic trees were visualized using ITOLv6 (50). All non-tree figures were created using ggplot2 in R and arranged using the ggpubr packages (46, 51). All maps were made using ggplot2 and rnaturalearthdata packages (52).

## RESULTS

**Fig 1:**
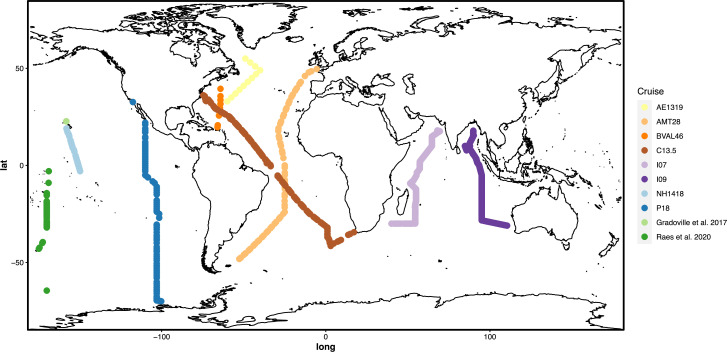
Global distribution of samples. Map of sampling locations for *nifH* amplicon sequences. Samples are grouped and colored based on region, using warm colors for the Atlantic Ocean, blue and green for the Pacific Ocean, and purple for the Indian Ocean samples.

**Fig 2:**
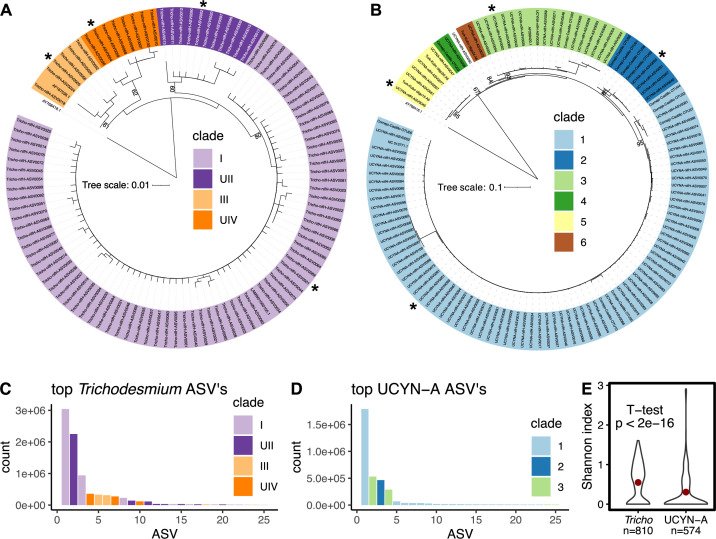
Phylogenetic tree of top 100 ASVs in *Trichodesmium* (**A**) and UCYN-A (**B**). Most abundant ASV for each clade is denoted by an *. Bootstrap values out of 100 are displayed at each node. ASV naming scheme is from most abundant to least abundant. Environmental counts of the top 25 ASV in *Trichodesmium* (**C**) and UCYN-A (**D**). Shannon diversity of *Trichodesmium* and UCYN-A (**E**). Mean value shown by red dot (*Trichodesmium* mean = 0.55 and UCYN-A mean = 0.30).

In order to capture the global distributions and environmental drivers of *Trichodesmium* and UCYN-A, we collected 954 surface ocean water samples as part of Bio-GO-SHIP and sequenced the nitrogenase gene *nifH* (Fig. 1). We combined this novel data set with previously generated samples collected during March 2014 at Stn. ALOHA (8) and samples collected during April 2016 until June 2016 on the P15S GO-SHIP line (26), resulting in a collection of 1102 samples (8, 26). *Trichodesmium* and UCYN-A accounted for over 95% of photoautotrophic *nifH* reads; therefore, we characterized the phylogeny of the top 100 most abundant sequences for each species and grouped them into existing major clades of *Trichodesmium* and UCYN-A (Fig. 2; Fig. S1 at https://doi.org/10.5281/zenodo.15682628). We then explored the global distributions of our sequences and linked these distributions to environmental measurements to determine the niche space of each clade.

Phylogenetic analysis of the *nifH* sequences captured known diversity as well as introduced novel representatives. We identified the 100 most abundant ASVs across all our samples. These ASVs constituted 98% of total *Trichodesmium* sequences and 94% of total UCYN-A sequences. Thus, we focused on characterizing these sequences further. We created phylogenetic trees from our most abundant sequences and contextualized them using known *nifH* sequences (Fig. 2A and B). *Trichodesmium* has previously characterized *nifH* sequences for only two of its four clades. Our tree reconstructed four distinct clades of *Trichodesmium*, which were present in 60%–80% of our tree bootstraps (Fig. 2A). Because two of the clades are based on *hetR* and internal transcribed spacer (ITS) sequences and have no previously characterized *nifH* sequences, we inferred the clades based on their proximity to clade I and III. We named these clades UII and UIV since they appear to match the already described clades II and IV, respectively. Of the top 100 most abundant ASVs corresponding to *Trichodesmium*, 75 corresponded to the clade *Tricho* I, 11 to *Tricho* UII, 6 to *Tricho* III, and 8 to *Tricho* UIV. UCYN-A has previously characterized *nifH* sequence representatives for each of its clades, and our tree captured the four major clades with high confidence (82%–95% of bootstraps). Of the top 100 most abundant ASVs corresponding to UCYN-A, 72 corresponded to UCYN-A1, 4 to UCYN-A2, 16 to UCYN-A3, 1 to UCYN-A4, 5 to UCYN-A5, 1 to UCYN-A6, and 1 did not fall within any known clade. Of the top 100 ASVs from *Trichodesmium* and UCYN-A, only a few ASVs made up the majority of acquired sequences (Fig. 2C and D). The most abundant *Trichodesmium* ASV, from *Tricho* I, made up 35% of sequences alone. Although many ASVs were associated with *Tricho* I, as seen in Fig. 2A, only two ASVs were highly abundant (Fig. 2C). The second most abundant ASV in *Trichodesmium* was part of clade *Tricho* UII and made up 26% of total sequences. The rest of the ASVs within the top 100 most abundant each represented <5% of the total sequences. Similarly, one ASV within UCYN-A1 made up 45% of all UCYN-A sequences. The second most abundant ASV fell within UCYN-A3 (13%), the third within UCYN-A2 (12%), and the fourth within UCYN-A3 (7.2%). Afterward, there was a sharp decline in the abundance of the remaining ASVs (<2% each). Clades UCYN-A4, UCYN-A5, UCYN-A6, and ASV 50 (which did not fall within a stable clade) combined only represented 0.54% of total sequences and thus were excluded from further analysis. When comparing the alpha diversity of *Trichodesmium* and UCYN-A, we observed a significantly higher average Shannon index for *Trichodesmium* than UCYN-A (*Trichodesmium* mean = 0.55, UCYN-A mean = 0.30, *t*-test, *P* < 2e–16) (Fig. 2E). In sum, both *Trichodesmium* and UCYN-A demonstrated highly skewed abundance distributions with only 3–4 ASVs dominating across the global surface ocean.

**Fig 3:**
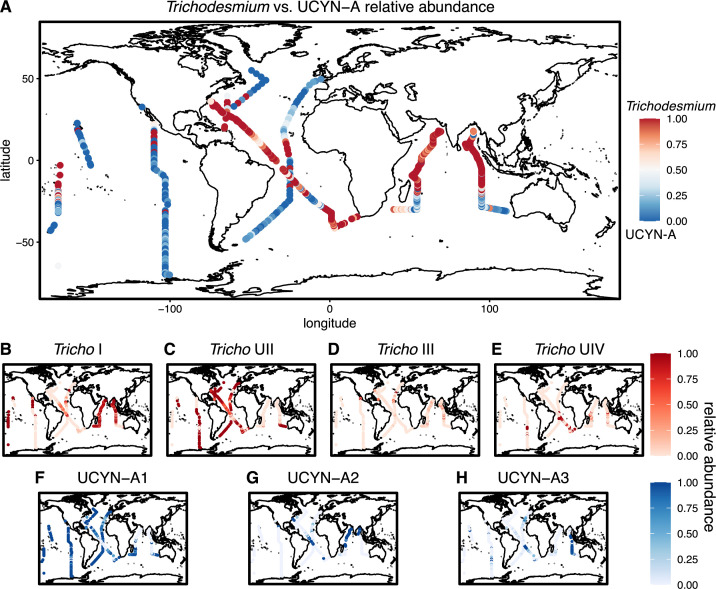
Biogeography of *Trichodesmium* and UCYN-A clades. Amplicon-derived relative abundance of *Trichodesmium* vs UCYNA-A (**A**). A value of 1 represents 100% *Tricho*, while a value of 0 represents 100% UCYN-A. Relative abundance of individual clades out of total *Trichodesmium* abundance (**B–E**). Relative abundance of individual clades out of total UCYN-A abundance (**F–H**).

The phylogeographies of *Trichodesmium* and UCYN-A reveal distinct regional differentiation between the different species and clades. When comparing the relative abundance of *Trichodesmium* vs UCYN-A, we see that *Trichodesmium* dominates in the northern Indian Ocean, while UCYN-A is more abundant across a majority of the Pacific Ocean (Fig. 3A). There is more variation between the two photoautotrophs across the Atlantic Ocean, as the AMT28 and C13.5 cruises show opposite patterns of dominance (Fig. 1 and 3A). This suggests that there may be variation between *Trichodesmium* and UCYN-A due to seasonality or longitudinal gradients. *Trichodesmium* I was abundant in warm regions with seasonally shallow nutricline depths, including the Indian Ocean and north of the equator in the Pacific and Atlantic Oceans (Fig. 3B). In contrast, the opposite pattern was observed in *Trichodesmium* UII, which was abundant in the southeastern Indian Ocean near Australia and in sub-tropical to sub-arctic regions throughout the Atlantic and Pacific Oceans (Fig. 3C). Clades I and II combined made up a majority of all *Trichodesmium* sequences (81.74% of total *Tricho* sequences; Fig. 3B and C). On the other hand, *Trichodesmium* III was abundant in the northern hemisphere of the Pacific and Atlantic Oceans, whereas *Trichodesmium* IV was abundant in the southern hemisphere (Fig. 3D and E). UCYN-A1 dominated throughout the globe (Fig. 3F). UCYN-A1 was the most abundant clade in samples where UCYN-A was more abundant than *Trichodesmium* (Fig. 3A and F). This clade was also dominated by a single ASV (UCYNA-*nifH*-ASV-0001; 76% of UCYN-A1 ASVs), resulting in a fairly homogeneous UCYN-A population across the globe. UCYN-A clades II and III were primarily found in the eastern and western Indian Ocean, respectively (Fig. 3G and H). Each of the clades examined demonstrated a unique, regionally partitioned biogeography. To further support these findings, we correlated the relative abundances based on amplicon sequences to relative abundances based on corresponding metagenomic sequences and found significant correspondence between the two methods (Spearman *R* = 0.5, *P* < 2.2e–16).

**Fig 4:**
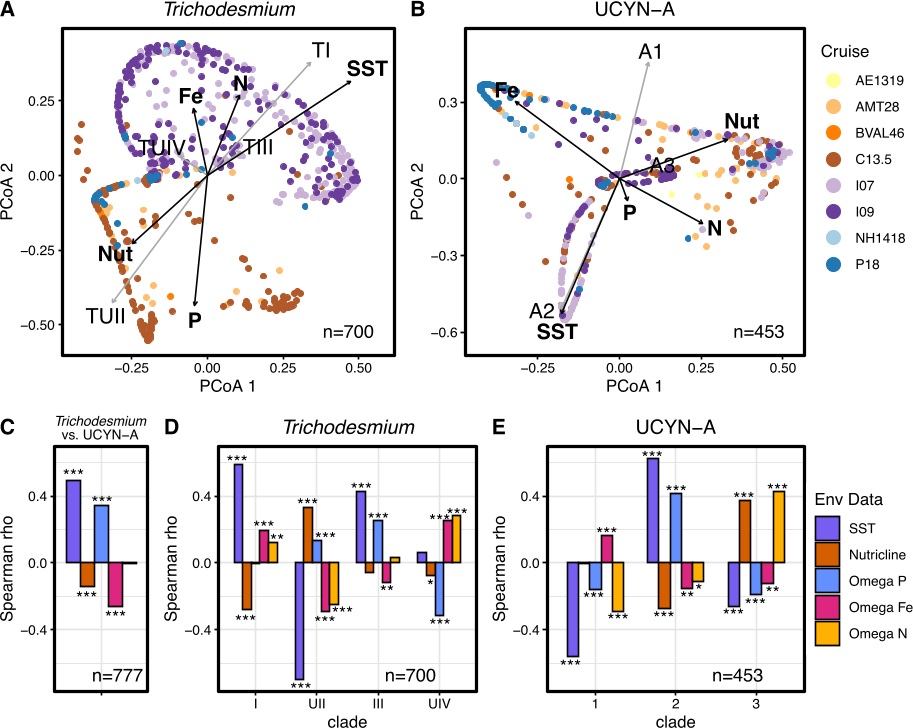
PCoA of *Trichodesmium* and UCYN-A beta-diversity (**A and B**). Correlations between environmental measurements (black arrows, bold label) and clade relative abundance (gray arrows, non-bolded label) and PCoA components. Exact values can be found in Tables S1 and S2 (**A and B**). Spearman correlations on the relative abundance of *Trichodesmium* and UCYN-A (**C**), *Trichodesmium* clade relative abundance (**D**), and UCYN-A clade relative abundance (**E**). Correlations are made with the following environmental data: sea surface temperature (SST), nutricline depth (nutricline), phosphorus stress (Ω P), iron stress (Ω Fe), and nitrogen stress (Ω N).

A comparison of environmental measurements revealed the factors shaping the composition of *Trichodesmium* and UCYN-A. A PCoA analysis of *Trichodesmium* beta-diversity revealed clear separation between ocean basins with the Atlantic Ocean (AMT28, BVAL46, and C13) and the Indian Ocean (I07 and I09) separating across the first two dimensions (PERMANOVA, *P* < 0.001, *R*^2^ = 0.10; Fig. 4A). We correlated environmental measurements to the PCoA results and found significant correlations for all factors compared to *Trichodesmium* (*P* < 0.0009). Sea surface temperature had the strongest correlation to *Trichodesmium* diversity (*R*^2^ = 0.32), followed by phosphorus stress (*R*^2^ = 0.19; Fig. 4A; Table S1 at https://doi.org/10.5281/zenodo.15682628). UCYN-A diversity did not partition based on ocean basin (Fig. 4B, Table S2). All environmental factors had a significant correlation with UCYN-A diversity (*P* < 0.0009) except for phosphorus stress. Temperature (*R*^2^ = 0.31), iron stress (*R*^2^ = 0.19), and nutricline depth (*R*^2^ = 0.13) explained the most variance (Fig. 4B; Table S2 at https://doi.org/10.5281/zenodo.15682628). The samples are clearly partitioned across these three factors in the PCoA and show overlap between the Atlantic and Indian Ocean, unlike *Trichodesmium*. A comparison of environmental factors to diversity revealed that *Trichodesmium* and UCYN-A diversity are both driven by different conditions.

We next compared the environmental drivers of *Trichodesmium* and UCYN-A microdiversity. For *Trichodesmium*, we observed a significant positive correlation between sea surface temperature and phosphorus stress. In contrast, UCYN-A aligned with a deep nutricline and iron stress (Fig. 4C; Table S3 at https://doi.org/10.5281/zenodo.15682628). The two most abundant *Trichodesmium* clades I and UII had opposite associations with environmental factors (Fig. 4D; Table S3). Clade I was more common under high temperatures, shallow nutriclines, iron stress, and nitrogen stress, while clade UII was associated with lower temperatures, deeper nutriclines, and phosphorus stress (Fig. 4D; Table S3). Clade III was associated with high temperatures and phosphorus stress (Fig. 4D; Table S3). Clade IV was mainly driven by nutrient conditions with a positive association with iron and nitrogen stress and a negative association to phosphorus stress (Fig. 4D; Table S3). The globally dominant UCYN-A1 clade had a significant negative association with sea surface temperature as well as significant correlations with all nutrient stress indicators (Fig. 4E; Table S3). UCYN-A2 and A3 are found in opposite parts of the Indian Ocean, which is reflected in their environmental drivers (Fig. 3G and H). Clade A2 is positively correlated to high temperatures and phosphorus stress found in the northern Indian Ocean, with A3 correlating to shallow nutriclines and nitrogen limitation (Fig. 4E; Table S3). Thus, different environmental factors are driving the global distributions and clades of both *Trichodesmium* and UCYN-A.

## DISCUSSION

When comparing the distributions and diversity of *Trichodesmium* and UCYN-A, we observed greater levels of microdiversity in *Trichodesmium* than in UCYN-A. This is likely due to differences in ecological strategy. *Trichodesmium* is a free-living cyanobacterium, directly influenced by variable environmental conditions that result in region-specific selection. On the other hand, UCYN-A is an obligate symbiont with small eukaryotic phytoplankton, giving it more consistent environmental conditions (24, 53). The limited diversity and dominance of a single UCYN-A ASV suggest it experiences stronger selective pressures, supporting the hypothesis that it is becoming integrated as an organelle (24). Alternatively, the *nifH* gene may be too conserved to capture the underlying microdiversity within UCYN-A. Overall, *Trichodesmium* diversity is separated by ocean basin (Fig. 3A), while UCYN-A populations have more overlap between regions. We observed a significant correlation of *Trichodesmium* vs UCYN-A distributions separated by sea surface temperature, Fe, and P nutrient stress. The global relationship with temperature aligns with previous quantitative polymerase chain reaction (qPCR)-based assessments showing a switching between *Trichodesmium* and UCYN-A, with lower temperatures favoring UCYN-A and vice versa (54). The correlation with Fe and P nutrient stress is likely a result of the high iron requirement of the nitrogenase enzyme (55, 56). In particular, *Trichodesmium* requires more Fe than other diazotrophs due to its need for Fe for both photosynthesis and nitrogen fixation simultaneously (57), while UCYN-A lacks certain genes for photosynthesis, relying on its host for carbon supply and some biosynthetic precursors (32).

While *Trichodesmium* distributions have been studied extensively at the genus level, investigation into the biogeography of *Trichodesmium* clades has been limited (7, 9, 11, 12). Four main clades of *Trichodesmium* have been characterized primarily using the *hetR* and ITS genes (13, 14). We identified four distinct clades based on the *nifH* gene (Fig. 2A). Two of these clades have not been previously identified using *nifH*, and thus, we inferred, based on their position on the tree relative to known clades, that they corresponded to *hetR*/ITS-defined clades II and IV. While it is highly likely they are the same clades, this cannot be confirmed without complete genomes containing both genes. Additionally, despite the fact that *Trichodesmium* IMS101 is a common laboratory isolate used in experimental work from clade III, we showed that clades I and II were the most globally abundant, while clade III was the least abundant out of the four clades. The dominance of clade I has been shown regionally, but the global distribution of clade II has never been characterized (8, 10, 16–18). This result further highlights that the use of isolate studies based on IMS101 may misinform modeling studies. Our findings suggest that the understudied clade II may warrant further physiological characterization, given its widespread abundance.

The observed distributions of UCYN-A clades corroborated previous regional assessments. In particular, it has been shown that a single oligotype dominates the clade UCYN-A1 (21, 58). We showed that this single ASV is abundant across regions where UCYN-A outcompetes *Trichodesmium*, with a link to Fe limitation (Fig. 3A, F, and 4E). The UCYN-A2 clade was initially proposed as a coastally adapted strain but has been observed in the Arctic Ocean and other high-latitude waters (53, 59–61). We saw a slight association with coastal proximity but did not capture this clade in our high-latitude samples in the Southern Ocean (Fig. 3G). As in clade UCYN-A1, we observed a single dominant ASV in UCYN-A2 populations (Fig. 2D). UCYN-A3 was the only clade that switched between two main ASVs (Fig. 2D). This clade has been shown to be globally distributed but at low relative abundance compared to UCYN-A1 (21). We found that UCYN-A3 is the dominant clade in the eastern Indian Ocean. The distribution of UCYN-A3, along with a significant correlation to a deep nutricline and high N stress, suggests that it has a distinct niche among UCYN-A populations and is not merely maintained as a low-abundance subpopulation. While there are other clades in UCYN-A, we found that most of the globe was dominated by UCYN-A1, UCYN-A2, and UCYN-A3, which is consistent with previous assessments (21).

While *nifH* has classically been used to capture diazotroph diversity, there are some important caveats to consider when interpreting the results of this study. First, as with all amplicon-based sequencing, there is an unknown level of primer bias, potentially increasing the abundance of some ASVs while underrepresenting others (62). To quantify this bias, we compared our amplicon-based results with metagenomic results but found poor coverage in many of our metagenomic samples. Previous metagenomic studies also found *nifH* sequences in only ~2/3 of the samples, with roughly a third lacking any sequences (7). Together, this suggests that multiple marker genes, such as *nifH*, *nifD*, and *nifK*, may be needed for accurate quantification in metagenomes. Second, the *nifH* gene is not a single-copy gene and can be found in variable copy numbers depending on the organism (63). Additionally, *nifH* has prevalent pseudogenes and homologs in organisms that are not diazotrophs, making it an imperfect indicator of diazotroph presence (63). Nevertheless, cyanobacteria possess a particularly strong link between *nifH* and diazotroph function. Specifically, non-diazotrophic *Trichodesmium* lacks the *nifH* gene, meaning any *nifH Trichodesmium* sequences are representative of the diazotrophic species (64). While imperfect, *nifH* is clearly linked to diazotrophic function in both UCYN-A and *Trichodesmium*, and the historical prevalence and abundance of *nifH* amplicon data provide a wealth of contextual data for amplicon-based studies.

This study provides an analysis of the global diversity and distribution of the diazotrophic cyanobacteria *Trichodesmium* and nitroplast UCYN-A, revealing intricate patterns of phylogenetic and ecological differentiation. By leveraging high-throughput sequencing of the *nifH* gene across extensive oceanic samples, we have uncovered significant clade-specific correlations to environmental variables such as temperature, nutrient availability, and nutrient stress. Our findings highlight differences in biogeography and putative ecological strategies between these two diazotrophs. The dominant presence of specific *Trichodesmium* and UCYN-A clades in distinct oceanic regions underscores their ecological specialization and potential environmental role. The data presented here serve as a valuable resource for future studies aimed at understanding the functional implications of genetic diversity in marine photoautotrophic bacteria and provide a foundation for exploring their roles in biogeochemical processes under a changing climate. This work not only enhances our understanding of marine microbial biogeography but also emphasizes the need for continued research into the adaptive mechanisms that enable these photoautotrophic bacteria to thrive in diverse marine environments.

## Data Availability

All code and data used in this analysis can be found at https://zenodo.org/doi/10.5281/zenodo.12636629. ASV sequences, ASV counts, metagnomic counts, and associated metadata are included in Data S1 to S5 at https://doi.org/10.5281/zenodo.15682628. NifH amplicon, 16s amplicon, and metagenomic data isare available on NCBI under BioProject ID PRJNA656268.
